# Predictors and moderators of burden of care and emotional distress in first-episode psychosis caregivers: results from the GET UP pragmatic cluster randomised controlled trial

**DOI:** 10.1017/S2045796019000155

**Published:** 2019-04-10

**Authors:** J. Onwumere, C. Bonetto, A. Lasalvia, E. Miglietta, A. Veronese, F. Bellini, M. Imbesi, P. Bebbington, E Kuipers, M. Ruggeri, The GET UP Group

**Affiliations:** 1 Department of Psychology, King's College London, Institute of Psychiatry, Psychology & Neuroscience, London, England; 2 Section of Psychiatry, Department of Neurosciences, Biomedicine and Movement Sciences, University of Verona, Verona, Italy; 3 Unit of Psychiatry, Azienda Ospedaliera Universitaria Integrata, Verona, Italy; 4 Department of Neurosciences, University of Padova and Azienda Ospedaliera, Padua, Italy; 5 Department of Mental Health, Azienda USL Romagna, CMHC Riccione, Italy; 6 Department of Mental Health, Azienda USL, Piacenza, Italy; 7 Department of Psychiatry, University College London, London, England

**Keywords:** Carers, families, first-episode psychosis, psychosocial interventions, treatment moderators

## Abstract

**Aims:**

First-episode psychosis (FEP) is a major life event and can have an adverse impact on the diagnosed individual and their families. The importance of intervening early and providing optimal treatments is widely acknowledged. In comparison to patient groups, literature is scarce on identifying treatment predictors and moderators of caregiver outcomes. This study aimed to identify pre-treatment characteristics predicting and/or moderating carer outcomes, based on data from a multi-element psychosocial intervention to FEP patients and carers (GET-UP PIANO trial).

**Methods:**

Carer demography, type of family relationship, patient contact hours, pre-treatment carer burden, patient perceptions of parental caregiving and expressed emotion (EE) were selected, *a priori*, as potential predictors/moderators of carer burden and emotional distress at 9 months post treatment. Outcomes were analysed separately in mixed-effects random regression models.

**Results:**

Analyses were performed on 260 carers. Only patient perceptions of early maternal criticism predicted reports of lower carer burden at follow-up. However, multiple imputation analysis failed to confirm this result. For treatment moderators: higher levels of carer burden at baseline yielded greater reductions in carer emotional distress at follow-up in the experimental group compared with treatment as usual (TAU). Higher levels of perceived EE moderated greater reductions in carer reports of tension in experimental group, compared with TAU, at follow-up. In younger caregivers (<51 years old), there were greater reductions in levels of worry during the baseline to follow-up period, within the experimental group compared with TAU.

**Conclusion:**

The study failed to identify significant treatment predictors of FEP carer outcomes. However, our preliminary findings suggest that optimal treatment outcomes for carers at first episode might be moderated by younger carer age, and carers reporting higher baseline levels of burden, and where patients perceive higher levels of negative effect from caregivers.

## Introduction

Psychotic disorders affect several million people worldwide (Fleischhacker *et al*., 2014). The peak phase of first onset often falls during late adolescence and early adulthood (Kirkbride *et al*., 2012). Many close relatives, predominately parents, will assume informal caregiving roles that can often be long-term (Onwumere *et al*., 2008; Boydell *et al*., 2014; Lavis *et al*., 2015). The importance and value of carer support in psychosis has been extensively reviewed in the literature. The pattern of evidence highlights improved illness course (Norman *et al*., 2005), mortality rates (Revier *et al*., 2015), treatment outcomes (Stowkowy *et al*., 2012) and facilitated access to relevant services for individuals with family support, when compared with peers without (Jansen *et al*., 2015*a*).

Though many families will take on caregiving responsibilities and in many cases will live with their relative with psychosis (Garety and Rigg, 2001; Cotton *et al*., 2013; Ran *et al*., 2016), a large proportion will also report experiencing high levels of carer burden, social isolation and a poorer quality of life, as part of their role (Gupta *et al*., 2015; Poon *et al*., 2016; Sadath *et al*., 2017). Psychosis can impact negatively on carer health and wellbeing, and lead to feelings of loss, burnout, worry, shame, self-stigma and psychological distress, which are already firmly established soon after first onset (Addington *et al*., 2003; Patterson *et al*., 2005; McCann *et al*., 2011; Boydell *et al*., 2014; Onwumere *et al*., 2017). Approximately 30–40% of carers report clinical depression and other indicators of psychological distress and morbidity (Kuipers and Raune, 2000; Hayes *et al*., 2015; Jansen *et al*., 2015*b*) and reports of distress and burden can persist (Brown and Birtwistle, 1998; Lee *et al*., 2014; Poon *et al*., 2016).

### Caregiving relationships and outcomes

Caregiving relationships characterised by elevated criticism, hostility and intrusive behaviours, and commonly described as high expressed emotion (EE), are typically predictive of a poorer illness course and outcomes in psychosis, including higher rates of patient relapse and rehospitalisation (Bebbington and Kuipers, 1994). This is particularly evident with reports of criticism that can have different underlying predictors and correlates (Alvarez-Jimenez *et al*., 2010; Cechnicki *et al*., 2013). Carers reporting higher levels of patient-focused criticism are more inclined to blame their relative for their illness and perceive illness symptoms and related behaviours as something their relative could control, if they chose to (Bentsen *et al*., 1998; Barrowclough and Hooley, 2003; McNab *et al*., 2007; Vasconcelos *et al*., 2013).

Patient perceptions of negative caregiving relationships (i.e. perceived EE) are themselves also linked to poorer patient functioning and outcomes (Onwumere *et al*., 2009; Hesse *et al*., 2016), which are observable at first episode (Von Polier *et al*., 2014; Haidl *et al*., 2018).

Carer burden is complex and multi-dimensional, and we know that higher levels are positively linked with greater levels of carer distress and negative caregiving relationships (Raune *et al*., 2004). Carer burden is also influenced by several clinical and demographic factors that hitherto have included carer age, the type of caregiving relationship (e.g. being a parent carer *v.* other carers) and illness beliefs (Kuipers and Bebbington, 2005; Gonclaves-pereira *et al*., 2013; Patel *et al*., 2014). EE and burden are long-term risk factors for poorer illness outcomes (Bebbington and Kuipers, 1994). Hence, the inclusion of evidence-based psychosocial interventions for individuals with psychosis and their families in several treatment guidelines across the globe (Kreyenbuhl *et al*., 2010; National Institute for Health and Care Excellence (NICE), 2014; Galletly *et al*., 2016; Norman *et al*., 2017). Traditionally, the interventions integrate different components such as psychoeducation, problem-solving, emotional processing, each designed to facilitate a better understanding about psychosis, a more relaxed family atmosphere and greater use of adaptive coping strategies.

#### The current study

The predictors of outcome across treatment groups can provide valuable prognostic information by helping to clarify which participants will respond more favourably to treatment in general, whereas treatment moderators provide prescriptive information about optimal treatment selection. Although there are clinical benefits in establishing baseline predictors of overall treatment success, identifying treatment moderators (i.e. who will do better in which treatment) may have more important clinical and cost-effectiveness implications.

There is, however, a very limited evidence base on treatment predictors in carer populations in psychosis. Further, where there is available data, they are rarely based on epidemiological representative samples compared with controls, which invariably increases the risk of underestimating the complexities of treating families in real-world services. Likewise, the literature is also scarce on moderators of treatment outcomes in carers. Despite the value of identifying the subgroups of caregivers and the circumstances associated with the effectiveness of early multi-element psychosocial interventions for psychosis, there is, as yet, little information about moderators of outcome. These findings would be extremely relevant in order to clarify generalisability issues of the experimental intervention effectiveness. The present study aims to address this gap in the literature.

As part of the GET UP (Genetics, Endophenotypes, Treatment: Understanding early Psychosis), PIANO (Psychosis: early Intervention and Assessment of Needs and Outcome) multi-element psychosocial intervention cluster trial in first-episode psychosis (FEP) (Ruggeri *et al*., 2012), the current study sought to identify, among pre-treatment characteristics, predictors and moderators of caregiver burden and emotional distress as measured by the General Health Questionnaire-12 (GHQ-12) self-report screen for psychiatric disturbance at 9 months post baseline. It aimed to understand: (a) which caregivers’ characteristics, among pre-treatment variables at baseline, are associated with a better treatment response regardless of treatment type (*non-specific predictors*); and (b) which characteristics are associated with a better response defined in terms of reduced levels of carer burden and emotional distress to the specific treatment provided in the GET UP PIANO trial (*moderators*). Based on the existing literature, we hypothesised that, regardless of treatment, improvement in carer burden and emotional distress at 9 months would be associated with non-parental caregivers, fewer hours per week spent between carers and patients and patients’ greater perception of positive care from carers (Bebbington and Kuipers, 1994; Kuipers and Bebbington, 2005; Awad and Voruganti, 2008; Poon *et al*., 2016; Sadath *et al*., 2017). Given the lack of available information, no specific *a priori* hypotheses were offered about moderators; thus, moderator analyses will be exploratory and utilise the same set of variables analysed as predictors.

## Methods

### The GET UP PIANO trial

The GET UP PIANO trial (Ruggeri *et al*., 2012) is a large multi-centre randomised controlled cluster trial comparing an add-on multi-element psychosocial early intervention with ‘routine care’ for patients with FEP and their caregivers provided within Italian public general mental health services. It was designed to assess early multi-element psychosocial interventions in epidemiologically representative samples of patients and families treated in routine generic mental health settings. Of the 126 community mental health centres (CMHCs) located in two northern Italian regions (Veneto and Emilia-Romagna) and the urban areas of Florence, Milan and Bolzano, 117 (92.8%) participated, covering an area of 9 304 093 inhabitants. The assignment units (clusters) were the CMHCs, and the units of observation and analysis were patients and their families. The trial received approval by the ethics committees of the coordinating centre (Azienda Ospedaliera Universitaria Integrata di Verona) and each participating unit and was registered with ClinicalTrials.gov (NCT01436331). Full details on the protocol of the GET UP PIANO study and on the main findings of the GET UP PIANO trial are given elsewhere (Ruggeri *et al*., 2012, 2015).

### Participants

During the index period, all CMHCs participating in the GET UP PIANO trial were asked to refer potential cases of psychosis at first contact to the study team. Inclusion eligibility comprised patients aged 18–54 years; residence within specified CMHC catchment area; presence of at least one of the following: hallucinations, delusions, qualitative speech disorder, qualitative psychomotor disorder, bizarre or grossly inappropriate behaviour, or two of the following: loss of interest, initiative and drive, social withdrawal, episodic severe excitement, purposeless destructiveness, overwhelming fear or marked self-neglect (as rated by the World Health Organisation (WHO) Screening Schedule for Psychosis (WHO, 1992), and first contact with CMHC). Exclusion criteria comprised a 3-month or greater history of use of anti-psychotic medication for treatment of the same or similar mental health problem; presence of other mental health condition(s) due to general medical condition; other International Classification of Diseases-10 psychiatric diagnosis (apart from psychosis); moderate–severe learning disability confirmed by clinical functional assessment.

Across both study arms, patients meeting inclusion criteria were invited to undertake standardised assessments as soon as possible, once they achieved clinical stabilisation and provision of informed written consent. They were provided with information detailing the nature, scope and possible consequences of participation in the trial and informed that they could withdraw consent at any time. Patient participants were also asked to give consent for family member contact; family members who agreed to participate provided written informed consent. There were no inclusion or exclusion criteria for relatives, beyond that all eligible patient participants were required to provide consent to involve a key family member in the assessments. The data are based on one identified carer per household.

### Treatments

The experimental treatment consisted of a multi-element psychosocial intervention, adjunctive to routine care. It included the delivery of cognitive behavioural therapy (CBT) for psychosis to patients (Kuipers *et al*., 1998; Garety *et al*., 2008), and of psychosis-focused family intervention (FI) (Kuipers *et al*., 2002) to families, together with case management (Burns and Firn, 2002), involving both patients and their families. The family-based, psychosis-focused intervention treatments were provided by psychiatrists and psychologists in the participating teams. They had completed specialist therapy training and had their competencies assessed with a specified minimum threshold level required to offer treatment to patients. The interventions typically include psychoeducation, emotional support, coping strategies, problem solving, emotional processing and relapse prevention (crisis planning) components that vary according to the presenting needs and agreed goals. The interventions comprised an optimal 10–15 sessions (typically six sessions in the initial 3 months followed by monthly sessions over the remaining 6 months) delivered over a 9-month period. Interventions were for individual families and delivered in accordance with the Kuipers *et al*.’s (2002) evidence-based treatment manual. To support treatment fidelity and avoid therapy drift, therapists attended regular supervision with external therapy experts that included written session summaries. An independent team of raters assessed random samples of audiotaped therapy recordings against therapy checklist. Control arm CMHCs provided only treatment as usual (TAU), which, in Italy, comprises personalised outpatient psychopharmacological treatment and non-specific supportive clinical management by the CMHC (Ferrannini *et al*., 2014). FIs in TAU consisted of non-specific informal support sessions.

### Measures

#### Carers

Carer outcomes (i.e. burden and emotional distress) were assessed by the Involvement Evaluation Questionnaire (IEQ-EU, van Wijngaarden *et al*., 2000) and the GHQ-12 (Goldberg and Williams, 1988) at baseline (before treatment was initiated) and at 9-month follow-up, by independent researchers, blind to treatment allocation.

The IEQ-EU (van Wijngaarden *et al*., 2000) is a widely used measure of carer burden that taps broad domains of caregiving experience and easy to complete. It is a 31-item four subscale questionnaire. The subscales relate to the encouragement and care that the caregiver has to give to the patient (urging); to personal problems between patient and caregiver (tension); to the caregiver's worries (worrying); and burden and monitoring patients about their medication, sleep and any dangerous behaviours (supervision). All items are scored on a five-point Likert scale. Higher scores indicate greater burden of care as an overall scale and within each domain. The measure has been translated into several different languages and culturally validated, including for use with Italian populations. It is psychometrically sound with proven reliability and validity data (Van Wijngaarden *et al*., 2000). Across different studies, internal consistency ratings (Cronbach's *α*) across the separate subscales have ranged from 0.68 to 0.86 and for the total scores has been 0.87–0.90. The test–retest reliability ratings are at least at 0.70 (Van Wijngaarden *et al*., 2000).

The GHQ-12 (Goldberg and Williams, 1988) is a global, widely used and cross-culturally validated measure to screen and identify minor psychiatric disorders. Each item assesses the severity of a mental health problem over the past few weeks using a four-point Likert scale. Higher scores indicate more psychological(emotional) distress. Its application as a unidimensional measure of distress has been most common through multidimensional approaches and focus on the individual factors (Graetz, 1991). It has extensive, worldwide published data attesting its reliability and validity in different groups (e.g. Werneke *et al*., 2000; Chandra Kashyap and Kant Singh, 2017) including those from Italy (Politi *et al.*, 1994), which yielded Cronbach's *α* ratings of 0.81. In its original form, the GHQ-12 was 60 items that were subsequently reduced to 30 items, 24 items and then 12 items. The 12-item version yields comparable reliability ratings to longer forms and has good validation against standardised mental health interviews (Goldberg *et al*., 1997; Politi *et al*., 1994).

#### Patients

The 25-item Parental Bonding Instrument (Parker *et al*., 1979) measures an adult's retrospective account of the parenting they received up to the age of 16 years. The measure is completed separately for care received from the mother and father. It yields two scales: ‘care’ and ‘overprotection’ (or ‘control’). Higher scores reflect a greater recollection of that parenting style. Optimal parenting is typically expressed by participant reports of high care and low control.

The Level of Expressed Emotion Scale (LEE, Cole and Kazarian, 1988; Cole and Kazarian, 1993) is a 60-item self-report measure designed to assess patient perceptions of carer EE. It was originally conceived as a reliable and expedient alternative to the Camberwell Family Interview (Vaughn and Leff, 1976), the gold standard measurement of carer EE. It comprises four subscales: emotional response (e.g. high emotional response to illness (e.g. anger)), negative attitude (e.g. doubt patient is genuinely ill, blame patient for illness), intrusiveness (e.g. offering unsolicited often critical advice and frequent attempts to have contact) and low tolerance and high expectations (e.g. intolerance of illness behaviour and impairments). Respondents are required to read through a set of brief statements and indicate to what degree the statement accurately represents their carer's behaviour towards them during the preceding 3 months on a Likert scale of 1 (untrue) to 4 (true). An overall EE and subscale scores are generated.

As a global measure of patient symptomatology, the Positive and Negative Syndrome Scale (PANSS, Kay *et al.*, 1987) was used. The PANSS is a 30-item semi-structured interview used to rate psychotic symptomatology and comprises three subscales related to positive symptoms, negative symptoms and general psychopathology. Interview items are rated on a seven-point Likert scale that reflects increasing levels of psychopathology with higher scores indicating higher levels of symptomatology.

The Childhood Experience of Care and Abuse Questionnaire (CECAQ, Bifulco *et al*., 1994) is a self-report questionnaire that taps adverse childhood experiences including reports of physical and sexual abuse and neglect. A single item that assesses patient perceptions of caregiver criticism was used as an additional method to assess relationship quality.

Before starting the assessments, independent evaluators received formal training in the use and administration of instruments, with measurement of their knowledge, skills and assessment of inter-rater reliability to assess competency. Assessments followed once patients had achieved clinical stabilisation (i.e. sufficient mental state stability to engage in a brief clinical assessment), provided written informed consent and prior to commencement of interventions.

### Statistical analyses

Analyses were conducted using an intention-to-treat approach. IEQ-EU and GHQ-12 scores were analysed separately in mixed-effects random regression models. In order to take into account the trial design in which caregivers (level 1) were nested within CMHCs (level 2) (CONSORT guidelines for cluster randomised trials; Campbell *et al*., 2012), the individual CMHCs were included in the models as a random effect. In order to identify predictors and moderators of treatment outcome according to MacArthur's approach (Kraemer *et al*., 2002), we selected, *a priori*, on clinical or empirical grounds and derived from the literature, pre-treatment caregivers’ variables. Specifically, we investigated age and gender of caregiver, family relationship shared with patient (parents *v*. others), hours per week spent with patient (<32 *v*. ⩾32), mother's criticism and father's criticism (assessed by CECA-Q item 6; yes *v*. no), PBI (care and protection (mother), care and protection (father)), LEE (emotional response, negative attitude, intrusiveness, tolerance and expectations) and IEQ-EU tension at baseline (this last variable considered only for GHQ-12). Each model included treatment allocation (*T* coded as +1/2 for caregivers in the Experimental Treatment Group and −1/2 for those in the TAU Group), one predictor/moderator (*M* standardised), their interaction (*T* × *M*) and the baseline score of the outcome investigated (*B* standardised). When the main effect of a variable was significant, but the interaction was not, the variable was considered a non-specific predictor of outcome. When the interaction was significant (regardless of the significance of main effects), the variable was considered as a moderator.

In a secondary analysis, missing data on outcomes were estimated using a multiple imputation approach by chained equations (MICE), which generate 50 different plausible imputed data sets and combines results from each of them. Multiple imputations by chained equations were applied because it enables different variable types to be handled; specifically, we used predictive mean matching to deal with possible non-normality when imputing continuous variables.

The *α* level was set to 0.05 for all main effects and interactions. No correction for multiple testing was applied due to the exploratory nature of the study. All statistical analyses were carried out using the STATA software package, version 13 (Stata Corp, 2013)

## Results

**Fig. 1.**
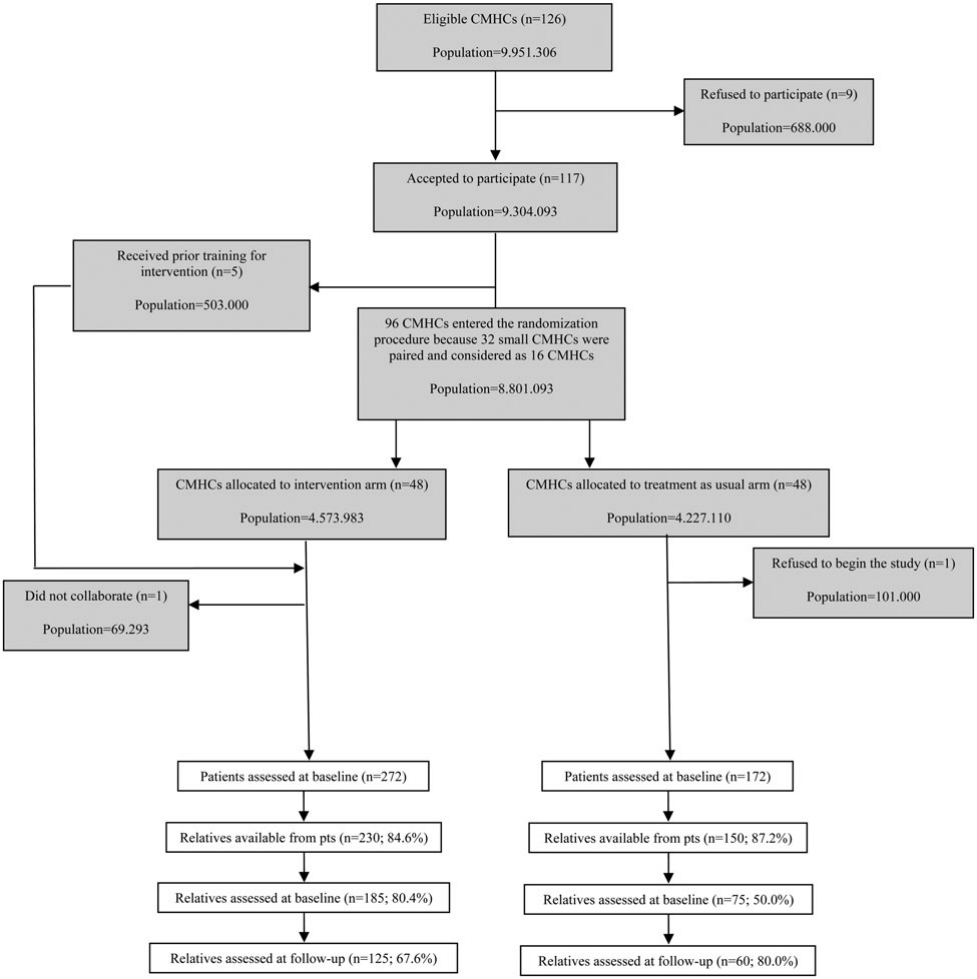
Trial profile for relatives.

Overall, 380 relatives (230 experimental; 150 TAU) out of 444 FEP patients were available for assessment at baseline. In the experimental arm, 16 patients did not have an identified relative; six patients declined consent to contact their relative; seven relatives declined consent to engage in the FI; and 13 patients refused to engage with the individual CBT, so the matched relative was excluded. In the TAU arm, ten patients did not have an identified relative and 12 patients declined consent to contact their relative (see Fig. 1 for relative's trial profile).

At baseline, 185 experimental arm and 75 TAU arm relatives were assessed. Demographic and pre-treatment characteristics of the 260 caregivers examined as potential predictors or moderators of outcome are presented in Table 1 and have been previously published elsewhere (Lasalvia *et al*., 2017; Ruggeri *et al*., 2017).

**Table 1.** Pre-treatment characteristics of caregivers examined as potential predictors/moderators of carer treatment outcome (EXP *n* = 185; TAU *n* = 75)

	BASELINE		*p*-value (*χ*^2^ or *t*-test, where appropriate)
	Treatment as usual group (*n* = 75)	Experimental treatment group (*n* = 185)	
**Gender, *n* (%)**			
Male	28 (37.3%)	69 (37.3%)	0.980
Female	47 (62.7%)	115 (62.7%)	
**Age, mean (s.d.)**	50.7 (10.5)	49.6 (11.2)	0.466
**Relationship with patient, *n* (%)**		(2 missing)	
Mother/father	47 (62.7%)	121 (66.1%)	0.597
Other	28 (37.3%)	62 (33.9%)	
**Patient's gender, *n* (%)**		(1 missing)	
Male	47 (62.7%)	113 (61.4%)	0.851
Female	28 (37.3%)	71 (38.6%)	
**Hours per week in the last 4 weeks, *n* (%)**		(5 missing)	
<32	24 (32.0%)	54 (30.0%)	0.752
⩾32	51 (68.0%)	126 (70.0%)	
**PBI Parental Bonding, mean (s.d.**) Mother Care Over protection Father Care Over protection	(5 missing) 17.2 (3.6) 19.2 (4.4) 16.4 (3.3) 17.6 (4.6)	(11 missing) 17.0 (3.7) 18.6 (4.6) 16.6 (4.0) 17.2 (5.0)	0.585 0.318 0.737 0.567
**LEE Expressed Emotion Level, mean (s.d.**) Emotional Response Negative Attitude Intrusiveness Tolerance and Expectations	(4 missing) 6.6 (1.8) 7.7 (2.0) 7.1 (1.9) 7.2 (1.7)	(9 missing) 7.0 (1.75) 8.2 (1.6) 7.5 (2.3) 7.3 (2.1)	0.120 0.056 0.131 0.745
**Criticism (CECA-Q item 6), *n* (%)**			
Mother	(3 missing)	(14 missing)	0.677
Yes	26 (36.1%)	57 (33.3%)	0.434
No	46 (63.9%)	114 (66.7%)	
Father	(8 missing)	(19 missing)	
Yes	21 (31.3%)	61 (36.7%)	
No	46 (68.7%)	105 (63.3%)	

No significant differences with respect to socio-demographics of relatives and link with patient variables were found between the two trial arms. At follow-up, 60 (32.4%) caregivers in the experimental group and 15 (20.0%) in the TAU group dropped out from assessment. There were no significant differences in demographics and outcome variables at baseline between completers and non-completers, with exception only of the GHQ-12 total score in the experimental group (completers: 14.27 s.d. 6.00 *v*. non-completers: 16.39 s.d. 7.84; *p* = 0.044).

By considering burden of care (IEQ-EU), both groups had similar baseline scores (*t*-test; *p* > 0.05). Specifically, we observed the following scores: Total EXP 2.07 s.d. 0.69 *v*. TAU 1.98 s.d. 0.63; Tension EXP 1.70 s.d. 0.66 *v*. TAU 1.57 s.d. 0.50; Supervision EXP 1.75 s.d. 0.99 *v*. TAU 1.58 s.d. 0.79; Worrying EXP 2.81 s.d. 1.15 *v*. TAU 2.69 s.d. 0.98; Urging EXP 2.09 s.d. 0.85 *v*. TAU 2.10 s.d. 0.89. Both groups experienced an improvement at follow-up, however no dimension reached statistical significance (Total EXP 1.79 s.d. 0.93 *v*. TAU 1.80 s.d. 0.64; Tension EXP 1.60 s.d. 1.02 *v*. TAU 1.58 s.d. 0.64; Supervision EXP 1.54 s.d. 1.13 *v*. TAU 1.38 s.d. 0.71; Worrying EXP 2.14 s.d. 0.96 *v*. TAU 2.31 s.d. 1.12; Urging EXP 1.81 s.d. 1.02 *v*. TAU 1.88 s.d. 1.02). Emotional distress (GHQ-12) differed significantly between the two groups at baseline (EXP 15.06 s.d. 6.82 *v*. TAU 12.97 s.d. 5.69; *p* = 0.023 *t*-test), while both groups experienced significant improvement at the 9 months follow-up – this proved more so for the experimental group (see Table 2) (EXP 10.88 s.d. 4.58 *v*. TAU 11.65 s.d. 6.03; (FU-BL) EXP *v*. TAU −1.71, *p* = 0.029).

**Table 2.** Relatives’ outcomes: IEQ and GHQ assessed at baseline and at 9-month follow-up, together with regression coefficients of experimental treatment *v*. treatment as usual (95% CI)

RELATIVES’ OUTCOMES	Treatment as usual group		Experimental treatment group		Regression coefficient^#^ of experimental treatment *v*. treatment as usual (95% CI)	*p*-value
	BL (*n* = 75)	FU (*n* = 60)	BL (*n* = 185)	FU (*n* = 125)		
		(2 missing)				
**IEQ total**	1.98 (0.63)	1.80 (0.64)	2.07 (0.69)	1.79 (0.93)	−0.03 (-0.30 to 0.24)	0.826
Tension	1.57 (0.50)	1.58 (0.64)	1.70 (0.66)	1.60 (1.02)	−0.05 (−0.35 to 0.24)	0.721
Supervision	1.58 (0.79)	1.38 (0.71)	1.75 (0.99)	1.54 (1.13)	0.10 (−0.22 to 0.42)	0.529
Worrying	2.69 (0.98)	2.31 (1.12)	2.81 (1.15)	2.14 (0.96)	−0.17 (−0.47 to 0.13)	0.279
Urging	2.10 (0.89)	1.88 (1.02)	2.09 (0.85)	1.81 (1.02)	−0.05 (−0.35 to 0.25)	0.740
		(2 missing)	(9 missing)	(3 missing)		
**GHQ**	12.97 (5.69)^a^	11.65 (6.03)	15.06 (6.82)^a^	10.88 (4.58)	−1.71 (-3.24 to -0.17)	0.029

a *p* = 0.023, *t*-test.

Most families in the experimental group engaged in at least one family session (91.1%, *n* = 170); the majority receiving five or more FI sessions (90.6%, *n* = 154), and from these, 72.7% (*n* = 112) attended ten or more sessions.

### Predictors

Of the predictors examined, only patient reports of early maternal criticism (i.e. during the first 16 years) predicted lower caregiver worrying as measured by IEQ-EU at 9 months (*b* = –0.36, *p* = 0.019), regardless of treatment assignment (see Table 3 Main effect column). However, multiple imputation analysis did not confirm this result.

**Table 3.** Pre-treatment characteristics as potential predictors/moderators of treatment outcome in caregivers. Mixed-effects random regression models estimated on caregivers who were assessed at both baseline and follow-up (EXP *n* = 125; TAU *n* = 60) (only variables significant at *p* < 0.05) are shown)

Potential predictor/moderator (pre-treatment)	Outcome at FU (adjusted for BL)	Main effect (prediction) *b* (95% CI), *p*	Interaction with treatment (moderation) *b* (95% CI), *p*
Age of caregiver	IEQ Worrying	+0.02 (-0.13; +0.16), *p* = 0.806	+0.35 (+0.06; +0.64), *p* = 0.017^*^
Mother's criticism	IEQ Worrying	−0.36 (-0.67;-0.06), *p* = 0.019	+0.53 (-0.07; +1.14), *p* = 0.085
IEQ Tension	GHQ	+0.21 (+0.01; +0.41), *p* = 0.036	−0.37 (-0.72;-0.01), *p* = 0.044*
LEE Tolerance and Expectations	IEQ Tension	+0.06 (-0.15; +0.26), *p* = 0.590	+0.48 (+0.07; +0.88), *p* = 0.021*

^*^Predictors/moderators which remained significant (*p* < 0.05) after applying multiple imputation procedure by chained equations (MICE).

### Moderators

Differential effects of pre-treatment IEQ-EU Tension on GHQ-12 (*b* = –0.37, *p* = 0.044) were found (see Table 2 Interaction with treatment column). Moreover, the LEE tolerance and expectations dimension moderated IEQ-EU Tension domain (*b* =  + 0.48, *p* = 0.021), while age of caregiver was a moderator of IEQ-EU Worrying (*b* =  + 0.35, *p* = 0.017). When analyses were rerun using multiple imputation of missing data, all these findings were confirmed (*b* = –0.38, *p* = 0.003; *b* =  + 0.42, *p* = 0.034 and *b* =  + 0.34, *p* = 0.022, respectively).

**Fig. 2.**
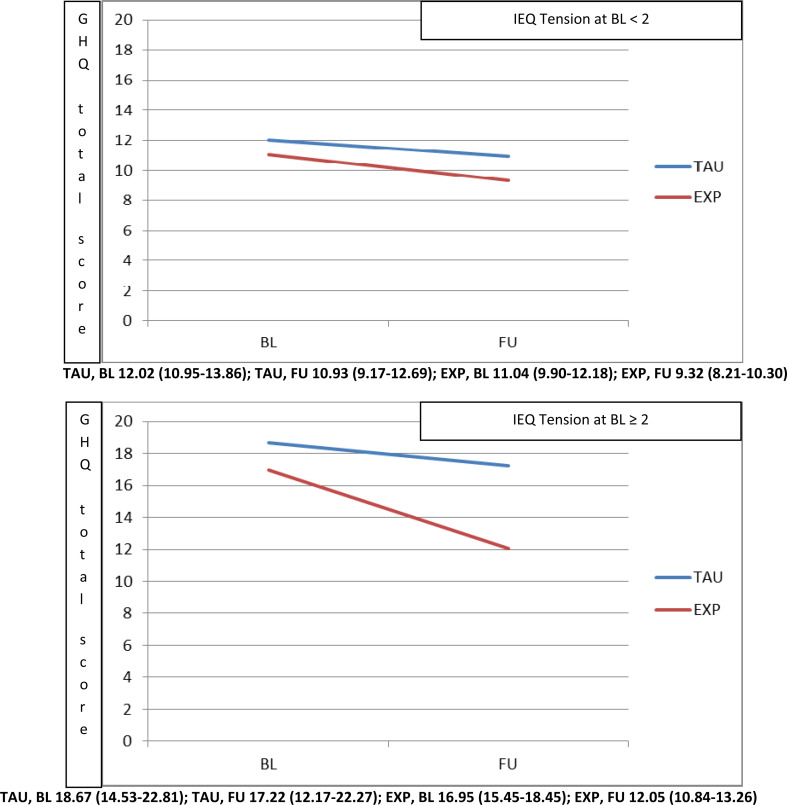
Moderation played by pre-treatment IEQ tension domain (top panel <2, bottom panel ⩾2) on the effect of intervention (Experimental *v*. TAU) on the GHQ-12 total score.

In order to determine the pre-treatment IEQ-EU Tension level cut-off at which the experimental treatment started to be significantly superior to usual care, the domain was categorised using different cut-offs in a sensitivity analysis. This analysis showed that starting from 2.0 there was a significantly higher beneficial effect of experimental treatment at 9 months, in terms of reduction in GHQ-12 total scores (Fig. 2). Carers with IEQ-EU Tension levels below 2 showed similar reduction of GHQ-12 in both experimental and usual treatment.

**Fig. 3.**
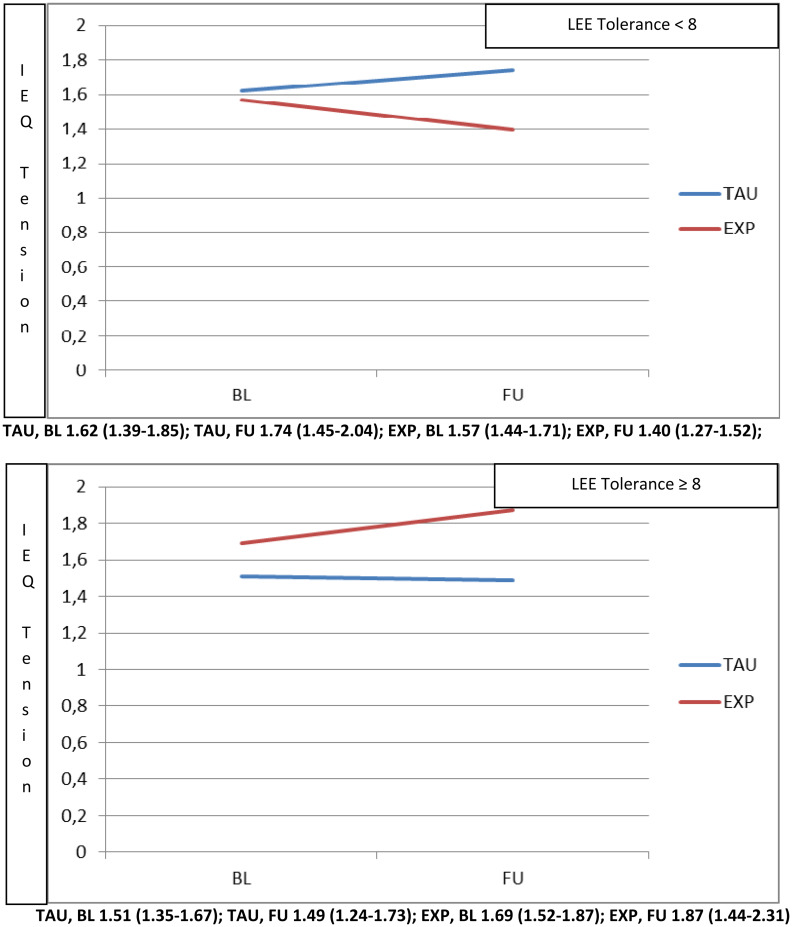
Moderation played by LEE tolerance and expectations domain (top panel <8, bottom panel ⩾8) on the effect of intervention (Experimental *v*. TAU) on the IEQ tension domain.

The same approach was applied in order to explore the moderation due to the LEE tolerance and expectations domain on IEQ-EU Tension. We found that where patients reported LEE tolerance and expectations levels below 8 (i.e. where patient perceptions of carer tolerance towards the patient were low), carers showed a significantly higher beneficial effect of experimental treatment at 9 months, in terms of reduction in IEQ-EU Tension scores (Fig. 3, top panel).

**Fig. 4.**
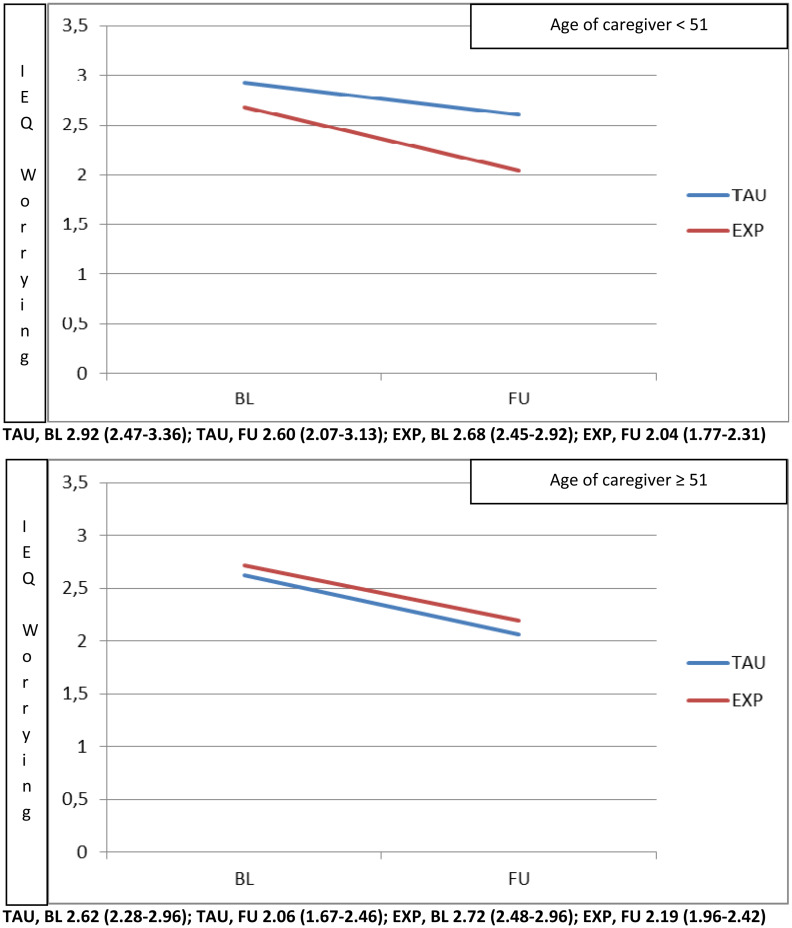
Moderation played by age of caregiver (top panel <51, bottom panel ⩾51) on the effect of intervention (Experimental *v*. TAU) on the IEQ worrying domain.

Finally, carers aged <51 years (at the top of Fig. 4) experienced a higher beneficial effect of experimental treatment in terms of reduction in IEQ-EU Worrying, while carers aged 51 years and above experienced at 9 months, a similar reduction of IEQ-EU Worrying in both the experimental and usual treatment arms.

## Discussion

The FEP can be a traumatic and stress-provoking period for individuals with psychosis and their families (McCann *et al*., 2011; Bendall *et al*., 2012). The illness course can fluctuate with elevated levels of relapse and poor social and vocational functioning (Robinson *et al*., 1999; Velthorst *et al*., 2017). The impact of care (i.e. carer burden) is often recorded at its highest levels during the first episode (Addington *et al*., 2003). Access to evidence-based psychosocial interventions, designed to improve understanding, uptake of adaptive coping and address the negative impact of illness on functioning and relationships, is increasingly proposed and implemented in several different countries (Mueser *et al*., 2015; Marwaha *et al*., 2016). This is the first study to investigate in a FEP ‘real-world’ setting which caregiver characteristics: (a) predict carer burden and emotional distress at 9 months regardless of treatment assignment (*non-specific predictors*) and (b) moderate differential response of treatment (*moderators*).

The results identified only one significant treatment predictor, which was patient perception of early maternal criticism. It predicted carer burden, specifically in terms of carer reports of worry. The significance of this finding, however, was not maintained after multiple imputation analysis for missing data. Thus, overall, the current findings did not identify pre-treatment predictors for carer outcomes. It is unclear why there was a failure to identify any significant predictors of treatment outcome for carers. It could be argued that the predictors, themselves, were not the most suitable. However, as reported, the selected predictors were drawn up based on indications from the relevant literature. The absence of significant findings suggest a greater complexity of factors potentially impacting carer treatment outcomes at FEP and highlighted the need for further work to isolate these key variables. It would seem important to note that it was only until very recently that family-based interventions recorded carer outcomes in their own right (Lobban *et al*., 2013), and highlighted the importance of looking at carer outcomes.

In contrast, our exploratory analyses identified three significant moderators of carer burden and distress. Higher pre-treatment levels of carer burden, specifically in terms of tension (i.e. strained and difficult relations between carer and relative), moderated effects of treatment on carer outcomes to yield greater reductions on emotional distress levels in carers. Patient perceptions of greater carer intolerance of the patient and their illness symptoms moderated greater reductions in carer burden in terms of tension; and younger age of caregiver (<51 years old) moderated greater reductions in carer burden, specifically in terms of worry. It could be suggested that carers expressing interpersonal difficulties with their relative which, in some circumstances, might have predated the psychosis onset, will also be the groups to derive the greatest benefits from the multi-component interventions. Whilst their elevated levels of burden could serve as a marker of those carers who are most in need and struggling with their understanding and adaptation to the illness. It could also simply be the case that given their elevated burden levels, there is more room to demonstrate improvements. However, the importance of not assuming that carers who present in a less overtly distressed manner or report less relationship difficulties with their relative do not require input from services is acknowledged (Treanor *et al*., 2013).

The uneven number of carers across the treatment conditions was noted. It possible that the relatives were more attracted by the description of the FI provided in the experimental arm as compared with the usual non-specific informal support sessions proposed in the control group. This phenomenon occurred on a naturalistic basis as all staff members received formal training in describing both treatments as efficacious.

The difficulties observed in the wellbeing and functioning of carers of long-term psychosis populations can typically emerge soon after the first episode. We know that family environment at FEP offers important implications for the quality and direction of patient outcomes (Domínguez-Martínez *et al*., 2014; Koutra *et al*., 2015; Haidl *et al*., 2018). Our results are encouraging and suggest multi-element psychosocial treatment approaches delivered during the FEP phase in routine mental health services, does appear to exert a specific and additional beneficial effect on caregivers (Penn *et al*., 2005), and we now have an awareness of potential factors that can moderate enhanced outcomes.

### Strengths and limitations

To the best of our knowledge, this study represents the first exploration of predictors and moderators of carer outcomes in FEP following multi-element treatments or TAU treatment. It extends similar work exploring general outcomes of psychosocial interventions in patients (Penn *et al*., 2005) and compliments developments in identifying treatment predictors and moderators in patients (Lasalvia *et al*., 2017). The sample size, prospective design methodology and rationale underpinning the study in a large catchment area and in a highly representative cohort of participants remain notable strengths. The study, however, does have limitations. First, the sample was drawn from specified Northern Central Italian regions, which means caution is required before generalising findings to groups from other socio-economic areas. The number of relatives that did not provide their consent to complete baseline assessments and the proportion of relatives classified as non-completers at follow-up might also serve as a limitation. Thus, it is possible that these relatives might have held specific appraisals regarding how they perceive their relative, the illness and the nature of their family relationship that limits the generalisability of findings to the wider group of carers. Second, we previously acknowledged that our moderator analyses were exploratory, with the primary aim of providing useful information for designing future studies. This is likely to improve with time following a greater focus on carer outcomes. We are aware that we performed a high number of statistical tests without correction. Multiple testing corrections are applied in order to reduce the number of false positives, but this correction may increase the number of false negatives, where there is an effect but we do not detect it as statistically significant. Due to the exploratory nature of this study, we did not apply multiple testing because the cost of a false negative could be that we have missed out on an important result to be confirmed in future larger studies.

### Implications

Our findings are encouraging but require replication and employment of samples drawn from other geographical contexts. Future considerations of the underlying mechanisms or key therapeutic components that give rise to the positive changes are indicated. We already know that in an unselected group of FEP carers in routine services, multi-element psychosocial interventions can yield more positive outcomes on carer distress and burden of care than TAU (Ruggeri *et al*., 2015). In services where resources might be limited and access to support triaged and prioritised, it would appear that younger aged carers exhibiting higher levels of burden, interpersonal difficulties with the patient and struggling to acknowledge that the identified patients do have a recognisable mental health problem that is likely to impact on their functioning and behaviour are also those most likely to exhibit the greatest gains from the interventions.

## Conclusion

Following the increasing and globalised focus on early intervention in psychosis (e.g. Marwaha *et al*., 2016), the results offer some helpful guidance on resource allocation and prioritisation. Though the evidence base for targeting recommended evidence-based interventions in psychosis to those identified to derive greatest benefit remains limited (Harvey*et al.*, 2018), our preliminary findings support the approach. The important role played by carers in helping to improve the scale and quality of patient outcomes in psychosis is well established, as is the need to provide comprehensive care packages to support them in their role (Mueser *et al*., 2015). However, far more evidence is required to improve our understanding of the benefits of interventions and key determinants of optimal carer outcomes in FEP.

## Availability of data and materials

The data that support the findings of this study are available, upon reasonable request and with permission, from Professor Mirella Ruggeri and The Get Up Group.

## Supplementary material

For supplementary material accompanying this paper visit https://doi.org/10.1017/S2045796019000155. click here to view supplementary material

## References

[ref1] Addington J, Coldham EL, Jones B, Ko T and Addington D (2003) The first episode of psychosis: the experience of relatives. Acta Psychiatrica Scandinavia 108, 285–289. 10.1034/j.1600-0447.2003.00153.x 12956829

[ref1a] Alvarez-Jiménez M, Gleeson JF, Cotton SM, Wade D, Crisp K, Yap MB, McGorry PD (2010) Differential predictors of critical comments and emotional over-involvement in first-episode psychosis. Psychological Medicine 40, 63–72. 19079825 10.1017/S0033291708004765

[ref2] Awad AG and Voruganti LNP (2008) The burden of schizophrenia on caregivers – a review. Pharmacoeconomics 26, 149–162. 18198934 10.2165/00019053-200826020-00005

[ref3] Barrowclough C and Hooley JM (2003) Attributions and expressed emotion: a review. Clinical Psychology Review 23, 849–880. 14529701 10.1016/s0272-7358(03)00075-8

[ref4] Bebbington P and Kuipers L (1994) The predictive utility of EE in schizophrenia: an aggregate analysis. Psychological Medicine 24, 707–718. 7991753 10.1017/s0033291700027860

[ref5] Bendall S, Alvarez-Jimenez M, Hulbert CA, McGorry PD and Jackson HJ (2012) Childhood trauma increases the risk of posttraumatic stress disorder in response to first-episode psychosis. Australian and New Zealand Journal of Psychiatry 46, 35–39. 10.1177/0004867411430877 22247091

[ref6] Bentsen H, Notland TH, Boye B, Munkvold OG, Bjorge H, Lersbryggen AB, Uren G, Oskarsson KH, Berg-Larsen R, Lingjaerde O and Malt UF (1998) Criticism and hostility in relatives of patients with schizophrenia or related psychoses: demographic and clinical predictors. Acta Psychiatrica Scandinavica 97, 76–85. 9504708 10.1111/j.1600-0447.1998.tb09967.x

[ref7] Bifulco A, Brown GW and Harris TO (1994) The childhood experience of care and abuse. Journal of Child Psychology and Psychiatry 35, 1419–1435. 7868637 10.1111/j.1469-7610.1994.tb01284.x

[ref8] Boydell J, Onwumere J, Dutta R, Bhavsar V, Hill N, Morgan C, Dazzan P, Morgan K, Pararajan M, Kuipers E, Jones P, Murray R and Fearon P (2014) Caregiving in first-episode psychosis: social characteristics associated with perceived ‘burden’ and associations with compulsory treatment. Early Intervention in Psychiatry 8, 122–129. 23458284 10.1111/eip.12041

[ref9] Brown S and Birtwistle J (1998) People with schizophrenia and their families. Fifteen-year outcome. British Journal of Psychiatry 173, 139–144. 10.1192/bjp.173.2.139 9850226

[ref10] Burns T and Firn M (2002) Assertive Outreach in Mental Health. A Manual for Practitioners. Oxford: Oxford University Press.

[ref11] Campbell MK, Piaggio G, Elbourne DR, Altman DG and CONSORT Group (2012) Consort 2010 statement: extension to cluster randomised trials. BMJ 345, e5661. 22951546 10.1136/bmj.e5661

[ref12] Cechnicki A, Bielańska A, Hanuszkiewicz I and Daren A (2013) The predictive validity of Expressed Emotions (EE) In schizophrenia. A 20-year prospective study. Journal of Psychiatric Research 47, 208–214. 23158233 10.1016/j.jpsychires.2012.10.004

[ref13] Chandra Kashyap G and Kant Sing S (2017) Reliability and validity of general health questionnaire (GHQ12) for male tannery workers: a study carried out in Kanpur, India. BMC Psychiatry 17, 102. 28320339 10.1186/s12888-017-1253-y PMC5360057

[ref14] Cole JD and Kazarian SS (1988) The level of expressed emotion scale. A new measure of expressed emotion. Journal of Clinical Psychology 44, 392–397. 3384966 10.1002/1097-4679(198805)44:3<392::aid-jclp2270440313>3.0.co;2-3

[ref15] Cole JD and Kazarian SS (1993) Predictive validity of the Level of Expressed Emotion (LEE) Scale: readmission follow-up data for 1, 2, and 5-year periods. Journal of Clinical Psychology 49, 216–218. 8098049 10.1002/1097-4679(199303)49:2<216::aid-jclp2270490214>3.0.co;2-g

[ref16] Cotton SM, McCann TV, Gleeson JF, Crisp K, Murphy BP and Lubman DI (2013) Coping strategies in carers of young people with first episode psychosis. Schizophrenia Research 146, 118–124. 23490761 10.1016/j.schres.2013.02.008

[ref17] Domínguez-Martínez T, Medina-Pradas C, Kwapil TR and Barrantes-Vidal N (2014) Relatives’ illness attributions mediate the association of expressed emotion with early psychosis symptoms and functioning. Psychiatry Research 218, 48–53. 24768246 10.1016/j.psychres.2014.04.012

[ref18] Ferrannini L, Ghio L, Gibertoni D, Lora A, Tibaldi G, Neri G, Piazza A and Italian Mental Health Data Group (2014) Thirty-five years of community psychiatry in Italy. The Journal of Nervous and Mental Disease 202, 432–439. 24821278 10.1097/NMD.0000000000000141

[ref19] Fleischhacker WW, Arango C, Arteel P, Barnes TRE, Carpenter W, Duckworth K, Galderisi S, Halper L, Knapp M, Marden SR, Moller M, Sartorius N and Woodruff P (2014) Schizophrenia – time to commit to policy change. Schizophrenia Bulletin 40(Suppl. 3),S165–S194 doi: 10.1093/schbul/sbu00. 24778411 PMC4002061

[ref20] Galletly C, Castle D, Dark F, Humberstone V, Jablensky A, Killackey E, Kulkarni J, McGorry P, Nielssen O and Tran N (2016) Royal Australian and New Zealand College of Psychiatrists clinical practice guidelines for the management of schizophrenia and related disorders. Australian and New Zealand Journal of Psychiatry 50, 410–472. 10.1177/0004867416641195 27106681

[ref21] Garety P and Rigg A (2001) Early psychosis in the inner city: a survey to inform service planning. Social Psychiatry and Psychiatric Epidemiology 36, 537–544. 11824848 10.1007/s001270170004

[ref22] Garety PA, Fowler DG, Freeman D, Bebbington P, Dunn G and Kuipers E (2008) Cognitive behavioural therapy and family intervention for relapse prevention and symptom reduction in psychosis: randomised controlled trial. British Journal of Psychiatry 192, 412–423. 10.1192/bjp.bp.107.043570 18515890

[ref23] Goldberg D and Williams P (1988) A User's Guide to the General Health Questionnaire. Windsor, UK: NFER-Nelson.

[ref24] Goldberg DP, Gater R, Sartorius N, Ustun TB, Piccinelli M, Gureje O and Rutter C. (1997) The validity of two versions of the GHQ in the WHO study of mental illness in general health care. Psychological Medicine 27, 191–197. 9122299 10.1017/s0033291796004242

[ref25] Gonclaves-pereira M, Xavier M, van Wijngaarden B, Papoila AL, Schene AH and Caldas-de-Almedia JM (2013) Impact of psychosis on Portuguese caregivers: a cross cultural explanation of burden, distress, positive aspects and clinical-functional correlates. Social Psychiatry and Psychiatric Epidemiology 48, 325–335. 22648702 10.1007/s00127-012-0516-7

[ref26] Graetz B (1991) Multidimensional properties of the General Health Questionnaire. Social Psychiatry and Psychiatric Epidemiology 26, 132–138. 1887291 10.1007/BF00782952

[ref27] Gupta S, Isherwood G, Jones K and Van Impe K (2015) Assessing health status in informal schizophrenia caregivers compared with health status in non-caregivers and caregivers of other conditions. BMC Psychiatry 15, 162. 26194890 10.1186/s12888-015-0547-1 PMC4509463

[ref27a] Haidl T, Rosen M, Schultze-Lutter F, Nieman D, Eggers S, Heinimaa M, Juckel G, Heinz A, Morrison A, Linszen D, Salokangas R, Klosterkötter J, Birchwood M, Patterson P, Ruhrmann S; European Prediction of Psychosis Study (EPOS) Group (2018) Expressed emotion as a predictor of the first psychotic episode - Results of the European prediction of psychosis study. Schizophrenia Research 199, 346–352. 29661524 10.1016/j.schres.2018.03.019

[ref28] Harvey C, Lewis J and Farhall J (2018) Receipt and targeting of evidence-based psychosocial interventions for people living with psychoses: findings from the second Australian national survey of psychosis. Epidemiology and Psychiatric Sciences. doi: 10.1017/S2045796018000288. PMC6998989 29893656

[ref29] Hayes L, Hawthorne G, Farhall J, O'Hanlon B and Harvey C (2015) Quality of life and social isolation among caregivers of adults with schizophrenia: policy and outcomes. Community Mental Health Journal 51, 591–597. 25690154 10.1007/s10597-015-9848-6

[ref30] Hesse K, Kriston L, Mehl S, Wittorf A, Wiedemann W, Wolwer W and Klingberg S (2016) The vicious cycle of family atmosphere, interpersonal self-concepts and paranoia in schizophrenia- a longitudinal study. Schizophrenia Bulletin 41, 1403–1412. 10.1093/schbul/sbv055 PMC4601709 25925392

[ref31] Jansen JE, Gleeson J and Cotton S (2015 *a*) Towards a better understanding of caregiver distress in early psychosis: a systematic review of the psychological factors involved. Clinical Psychology Review 35, 56–66. 25531423 10.1016/j.cpr.2014.12.002

[ref32] Jansen JE, Haahr UH, Harder S, Trauelsen AM, Lyse HG, Pedersen MB and Simonsen E (2015 *b*) Caregiver distress in first-episode psychosis: the role of subjective appraisal, over-involvement and symptomatology. Social Psychiatry Psychiatric Epidemiology 50, 371–378. 25053150 10.1007/s00127-014-0935-8

[ref33] Kay SR, Fiszbein A and Opler LA (1987) The positive and negative syndrome scale for schizophrenia. Schizophrenia Bulletin 13, 261–276. 3616518 10.1093/schbul/13.2.261

[ref34] Kirkbride JB, Errazuriz A, Croudace TJ, Morgan C, Jackson D, McCrone P and Murray RM (2012) Systematic review of the incidence and prevalence of schizophrenia and other psychoses in England. Department of Health Policy Research Programme.

[ref35] Koutra K, Trilivac S, Roumeliotaki T, Basta M, Simos P, Lionis C and Vgontzas AN (2015) Impaired family functioning in psychosis and its relevance to relapse: a two-year follow-up study. Comprehensive Psychiatry 62, 1–12. 26343461 10.1016/j.comppsych.2015.06.006

[ref36] Kraemer HC, Wilson GT, Fairburn CG and Agras WS (2002) Mediators and moderators of treatment effects in randomized clinical trials. Archives of General Psychiatry 59, 877–883. 12365874 10.1001/archpsyc.59.10.877

[ref37] Kreyenbuhl J, Buchanan RW, Dickerson FB and Dixon LB (2010) The Schizophrenia Patient Outcomes Research Team (PORT): updated treatment recommendations 2009. Schizophrenia Bulletin 36, 94–103. 19955388 10.1093/schbul/sbp130 PMC2800150

[ref38] Kuipers E and Bebbington P (2005) Research on Burden and Coping Strategies in families of people with mental disorders: problems and perspectives. Chapter 10, pp. 217–234. In Sartorius N, Leff J, L'opez-Ibor JJ, Maj M & Okasha A (eds), Families and Mental Disorders: From Burden to Empowerment. London: John Wiley & Sons.

[ref39] Kuipers E and Raune D (2000) The early development of expressed emotion and burden in the families of first onset psychosis. In Birchwood M, Fowler D and Jackson C (eds), Early Intervention in Psychosis. Chichester: Wiley, pp. 128–140.

[ref40] Kuipers E, Fowler D, Garety P, Chisolm D, Freeman D, Dunn G, Bebbington P and Hadley C (1998) London-east Anglia randomised controlled trial of cognitive-behavioural therapy for psychosis. III: follow-up and economic evaluation at 18 months. British Journal of Psychiatry 173, 61–68. 10.1192/bjp.173.1.61 9850205

[ref41] Kuipers E, Leff J and Lam D (2002) Family Work for Schizophrenia: A Practical Guide. London: Gaskell.

[ref43] Lasalvia A, Bonetto C, Lenzi J, Rucci P, Lozzino L, Cellini M (2017) Predictors and moderators of treatment outcome in patients receiving multi-element psychosocial intervention for early psychosis. Results from the Get UP pragmatic cluster randomized controlled trial. The British Journal of Psychiatry 210, 342–349. 28302703 10.1192/bjp.bp.116.190058

[ref44] Lavis A, Lester H, Everard L, Freemantle N, Amos T, Fowler D, Hodgekins J, Jones P, Marshall M, Sharma V, Larsen J, McCrone P, Singh S, Smith J and Birchwood M (2015) Layers of listening: qualitative analysis of the impact of early intervention services for first episode psychosis on carers’ experiences. The British Journal of Psychiatry 207, 135–142. 25999336 10.1192/bjp.bp.114.146415

[ref45] Lee G, Barrowclough C and Lobban F (2014) Positive affect in the family environment protects against relapse in first-episode psychosis. Social Psychiatry and Psychiatric Epidemiology 49, 367–376. 24081324 10.1007/s00127-013-0768-x

[ref46] Lobban F, Postlethwaite A, Glentworth D, Pinfold V, Wainwright L, Dunn G, Clancy A and Haddock G (2013) A systematic review of randomised controlled trials of interventions reporting outcomes for relatives of people with psychosis. Clinical Psychology Review 33, 372–382. 23410719 10.1016/j.cpr.2012.12.004

[ref48] Marwaha S, Thompson A, Upthegrove R and Broome MR (2016) Fifteen years on- early intervention for a new generation. The British Journal of Psychiatry 209, 186–188. 27587758 10.1192/bjp.bp.115.170035

[ref49] McCann TV, Lubman DI and Clark E (2011) First-time primary caregivers’ experience of caring for young adults with first-episode psychosis. Schizophrenia Bulletin 37, 381–388. 19679716 10.1093/schbul/sbp085 PMC3044625

[ref50] McNab C, Haslam N and Burnett P (2007) Expressed emotion, attributions, utility beliefs and distress in parents of young people with first episode psychosis. Psychiatry Research 15, 97–106. 10.1016/j.psychres.2006.08.004 17376540

[ref51] Mueser KT, Penn DL, Addington J, Brunette MF, Gingerich S, Glynn SM, Lynde DW, Gottlieb JD, Meyer-Kalos P, McGurk SR, Cather C, Saade S, Robinson DG, Schooler NR, Rosenheck RA and Kane JM (2015) The NAVIGATE program for first episode psychosis: rationale, overview and description of psychosocial components. Psychiatry Services 66, 680–690. 10.1176/appi.ps.201400413 PMC4490051 25772766

[ref52] National Institute for Health and Care Excellence (2014) Psychosis and Schizophrenia in Adults: Treatment and Management. Clinical Guideline 178. London: NICE. 25340235

[ref53] Norman RMG, Malla AK, Manchanda R, Harricharan R, Takhar J and Northcott SS (2005) Social support and three-year symptom and admission outcomes for first episode psychosis. Schizophr Research 80, 227–234. 10.1016/j.schres.2005.05.006 15964175

[ref54] Norman R, Lecomte T, Addington D and Anderson E (2017) Canadian treatment guidelines on psychosocial treatment of schizophrenia in adults. Canadian Journal of Psychiatry/La Revue Canadienne de Psychiatrie 62, 617–623. 10.1177/0706743717719894 PMC5593243 28703017

[ref55] Onwumere J, Kuipers E, Bebbington P, Dunn G, Fowler D, Freeman D, Watson P and Garety P (2008) Care-giving and illness beliefs in the course of psychotic illness. Canadian Journal of Psychiatry 53, 460–468. 18674404 10.1177/070674370805300711

[ref56] Onwumere J, Kuipers E, Bebbington P, Dunn G, Freeman D, Fowler D and Garety P (2009) Patient perceptions of caregiver criticism in psychosis: links with patient and caregiver functioning. The Journal of Nervous and Mental Disease 197, 85–91. 19214042 10.1097/NMD.0b013e3181960e57

[ref57] Onwumere J, Lotey G, Schulz J, James G, Afsharzadegan R, Harvey R, Chu Man L, Kuipers E and Raune D (2017) Burnout in early course psychosis caregivers: the role of illness beliefs and coping styles. Early Intervention in Psychiatry 11, 237–243. 25721376 10.1111/eip.12227

[ref58] Parker G, Tupling H and Brown LB (1979) A parental bonding instrument. British Journal of Medical Psychology 52, 1–10.

[ref59] Patel M, Chawla R, Krynicki CR, Rankin P and Upthgrove R (2014) Health beliefs and carer burden in first episode psychosis. BMC Psychiatry 14, 171. doi: 10.1186/1471-244X-14-171. 24913656 PMC4094457

[ref60] Patterson P, Birchwood M and Cochrane R (2005) Expressed emotion as an adaptation to loss – prospective study in first-episode psychosis. British Journal of Psychiatry 187(Suppl. 48), S59–S64. 10.1192/bjp.187.48.s59 16055810

[ref61] Penn DL, Waldheter EJ, Perkins DO, Mueser KT and Lieberman JA (2005) Psychosocial treatment for first episode psychosis: a research update. American Journal of Psychiatry 162, 2220–2232. 10.1176/appi.ajp.162.12.2220 16330584

[ref62] Politi PL, Piccinelli M and Wilkinson G (1994) Reliability, validity and factor structure of the 12-item General Health Questionnaire among young males in Italy. Acta Psychiatrica Scandinavica 90, 432–437. 7892776 10.1111/j.1600-0447.1994.tb01620.x

[ref63] Poon AW, Harvey C, Mackinnon A and Joubert L (2016) A longitudinal population-based study of carers of people with psychosis. Epidemiology and Psychiatric Sciences 5, 1–11. 10.1017/S2045796015001195 PMC6998635 26847994

[ref64] Ran MS, Chui CHK, Wong IY-L, Mao WJ, Lin FR, Liu B and Chan CLW (2016) Family caregivers and outcome of people with schizophrenia in rural China: 14 year follow up study. Social Psychiatry and Psychiatric Epidemiology 51, 513–520. 26724945 10.1007/s00127-015-1169-0

[ref65] Raune D, Kuipers E and Bebbington P (2004) Expressed emotion at first episode psychosis: investigating a carer appraisal model. British Journal of Psychiatry 184, 321–326. 10.1192/bjp.184.4.321 15056576

[ref66] Revier CJ, Reininghaus U, Dutta R, Fearon P, Murray R, Doody GA, Croudace T, Dazzan P, Heslin M, Onyejiaka A, Kravariti E, Lappin J, Lomas B, Kirkbride JB, Donoghue K, Morgan C and Jones PB (2015) Ten-year outcomes of first-episode psychoses in the MRC AESOP-10 study. The Journal of Nervous and Mental Disease 203, 379–386. 25900547 10.1097/NMD.0000000000000295 PMC4414339

[ref67] Robinson D, Woerner MG, Alvir JM, Bilder R, Goldman R, Geisler S, Koreen A, Sheitman B, Chakos M, Mayerhoff D and Lieberman JA (1999) Predictors of relapse following response from a first episode of schizophrenia or schizoaffective disorder. Archives of General Psychiatry 56, 241–247. 10078501 10.1001/archpsyc.56.3.241

[ref68] Ruggeri M, Bonetto C, Lasalvia A, De Girolamo G, Fioritti A, Rucci P (2012) A multi-element psychosocial intervention for early psychosis (GET UP PIANO TRIAL) conducted in a catchment area of 10 million inhabitants: study protocol for a pragmatic cluster randomized controlled trial. Trials 13, 73. 22647399 10.1186/1745-6215-13-73 PMC3464965

[ref69] Ruggeri M, Bonetto C, Lasalvia A, Fioritti A, de Girolamo G, Santonastaso P (2015) Feasibility and effectiveness of a multi-element psychosocial intervention for first-episode psychosis: results from the cluster-randomized controlled GET UP PIANO trial in a Catchment Area of 10 Million Inhabitants. Schizophrenia Bulletin 4, 1192–1203. 10.1093/schbul/sbv058 PMC4535643 25995057

[ref70] Ruggeri M, Lasalvia A, Santonastaso P, Pileggi F, Leuci E, Miceli M (2017) Family burden, emotional distress and service satisfaction in first episode psychosis: a nine month follow up study comparing TAU and a multi element integrated intervention. Data from the Get UP Trial. Frontiers in Psychology 8, 721. 28559862 10.3389/fpsyg.2017.00721 PMC5432637

[ref71] Sadath A, Muralidhar D, Varambally S, Gangadhar BN and Jose JP (2017) Do stress and support matter for caring? The role of perceived stress and social support on expressed emotion of carers of persons with first episode psychosis. Asian Journal of Psychiatry 25, 163–168. 28262142 10.1016/j.ajp.2016.10.023

[ref72] Stata Corp (2013) Stata Statistical Software: Release 13. College Station, TX: StataCorp LP.

[ref73] Stowkowy J, Addington D, Liu L, Hollowell B and Addington J (2012) Predictors of disengagement from treatment in an early psychosis program. Schizophrenia Research 136, 7–12. 22336955 10.1016/j.schres.2012.01.027

[ref74] Treanor L, Lobban F and Barrowclough C (2013) Relatives responses to psychosis: an exploratory investigation of low expressed emotion relatives. Psychology and Psychotherapy 86, 197–211. 23674469 10.1111/j.2044-8341.2011.02055.x

[ref75] van Wijngaarden B, Schene AH, Koeter MA, Vazquez-Barquero JL, Knudsen HC, Lasalvia A, Mcrone P and the Epsilon Study Group (2000) Caregiving in schizophrenia: development, internal consistency and reliability of the involvement evaluation questionnaire – European version. British Journal of Psychiatry 177, s21–s27. 10.1192/bjp.177.39.s21 10945074

[ref76] Vasconcelos ESD, Wearden A and Barrowclough C (2013) Expressed emotion, types of behavioural control and controllability attributions in relatives of people with recent-onset psychosis. Social Psychiatry and Psychiatric Epidemiology 48, 1377–1388. 23407901 10.1007/s00127-013-0659-1

[ref77] Vaughn CE and Leff JP (1976) The measurement of expressed emotion in the families of psychiatric patients. British Journal of Social and Clinical Psychology 15, 157–165. 10.1111/j.2044-8260.1976.tb00021.x 938822

[ref78] Velthorst E, Fett AKJ, Reichenberg A, Perlman G, van OS J, Bromet EJ and Kotov R (2017) The 20 year longitudinal trajectories of social functioning in individuals with psychotic disorders. American Journal of Psychiatry 174, 1075–1085 10.1176/appi.ajp.2016.15111419. PMC5474222 27978770

[ref79] Von Polier GG, Meng H, Lambert M, Strauss M, Zarotti G, Karle M, Dubois R, Stark FM, Neidhart S, Zollinger R, Burgin D, Felder W, Resch F, Koch E, Schulte-Markwort M and Schimmelmann BG (2014) Patterns and correlates of expressed emotion, perceived criticism and rearing style in first admitted early onset schizophrenia spectrum disorders. The Journal of Nervous and Mental Disease 202, 783–787. 25259947 10.1097/NMD.0000000000000209

[ref80] Werneke U, Goldberg DP, Yalcin I and Ustun BT (2000) The stability of the factor structure of the General Health Questionnaire. Psychological Medicine 30, 823–829. 11037090 10.1017/s0033291799002287

[ref81] World Health Organisation (1992) Schedules for Clinical Assessment in Neuropsychiatry (SCAN), Version 1.0. Geneva: World Health Organisation.

