# Supplementary material for: Predictors and moderators of burden of care and emotional distress in first-episode psychosis caregivers: results from the GET UP pragmatic cluster randomised controlled trial
Source: Epidemiol Psychiatr Sci. 2019 Apr 10;29:e27. doi: 10.1017/S2045796019000155 (PMC8061200; doi:10.1017/S2045796019000155)
Supplement: Supplementary file 1 [file S2045796019000155sup001.docx]

**THE GET UP GROUP includes:**

**GET UP - Genetics, Endophenotypes, Treatment: Understanding early Psychosis**

***NationalCoordinator:Professor Mirella Ruggeri (Verona)***

**Leading Project: PIANO (P**sychosis: Early **I**ntervention and **A**ssessment of **N**eeds and **O**utcome)

**Scientific Coordinator:** Mirella Ruggeri (Verona)

**Administrative Leading Institution:** *Azienda Ospedaliera Universitaria Integrata Verona, Regione Veneto*

**Coordinating Centre:** Mario Ballarin, Maria Elena Bertani, Sarah Bissoli, Chiara Bonetto, Doriana Cristofalo, Katia De Santi, Antonio Lasalvia, Silvia Lunardi, Valentina Negretto, Sara Poli, Sarah Tosato, Maria Grazia Zamboni

**Project TRUMPET** (**TR**aining and **U**nderstanding of Service **M**odels for **P**sychosis **E**arly **T**reatment)

**Scientific Coordinator: Giovanni De Girolamo (Bologna and Brescia)**

**Administrative Leading Institution:** *Agenzia Sanitaria e Sociale Regionale, Regione Emilia Romagna*

Coordinating Centre: Angelo Fioritti, Giovanni Neri, Francesca Pileggi, Paola Rucci

**Project GUITAR (G**enetic data **U**tilization and **I**mplementation of **T**argeted Drug **A**dministration _in the Clinical **R**outine)

**Scientific Coordinator: Massimo Gennarelli (Brescia)**

**Administrative Leading Institution:** *IRCCS Centro S.Giovanni di Dio Fatebenefratelli, Brescia*

Coordinating Centre: Luisella Bocchio Chiavetto, Catia Scasselatti, Roberta Zanardini

**Project CONTRABASS**

**CO**gnitive **N**euroendophenotypes for **T**reatment and **R**eh**A**bilitation of psychoses: **B**rain imaging, Infl**A**mmation and Stre**SS**

**Scientific Coordinator: Paolo Brambilla (Udine and Verona)**

**Administrative Leading Institution:** *Azienda Ospedaliera Universitaria Integrata, Verona, Regione Veneto*

Coordinating Centre: Marcella Bellani, Alessandra Bertoldo, Veronica Marinelli, Cinzia Perlini, Gianluca Rambaldelli

**ENROLLMENT AND TREATMENT RESEARCH UNITS:**

**RESEARCH UNIT Western Veneto:**

**Coordinator:** Antonio Lasalvia (Verona).

**Administrative Leading Institution:** *Azienda Ospedaliera Universitaria Integrata, Verona.*

**Coordinating Centre**: Mariaelena Bertani, Sarah Bissoli, Lorenza Lazzarotto.

**Participating MHCs:** ***TAU Arm***: Ulss 3 (Bassano), Ulss 4 Alto Vicentino (Thiene), Ulss 5 Montecchio (Centro; Sud), Ulss 6 Vicenza ( Secondo), Ulss 18 Rovigo (Rovigo), Ulss 20 Verona ( II° Servizio), Ulss 22 Bussolengo (Isola della Scala).

***Experimental Arm***: Ulss 5 Montecchio (Nord), Ulss 6 Vicenza (Primo; Noventa), Ulss 18 Rovigo ( Badia), Ulss 19 Adria (Adria), Ulss 20 Verona (I° Servizio; III° Servizio; La Filanda),Ulss 21 Legnago (Il Tulipano; il Girasole).

**MHCs Reference Persons**: Sonia Bardella, Francesco Gardellin, Dario Lamonaca, Antonio Lasalvia, Marco Lunardon, Renato Magnabosco, Marilena Martucci, Stylianos Nicolau Francesco Nifosi, Michele Pavanati, Massimo Rossi, Carlo Piazza, Gabriella Piccione, Annalisa Sala, Benedetta Stefani, Spyridon Zotos.

**CBT Staff:** Mirko Balbo, Ileana Boggian, Enrico Ceccato, Rosa Dall’Agnola, Francesco Gardellin, Barbara Girotto, Claudia Goss, Dario Lamonaca, Antonio Lasalvia, Alessia Mai, Annalisa Pasqualini, Michele Pavanati, Carlo Piazza, Gabriella Piccione, Stefano Roccato, Alberto Rossi, Spyridon Zotos.

**Family Intervention Staff**: Flavia Aldi, Barbara Bianchi, Paola Cappellari, Raffaello Conti, Laura De Battisti, Silvia Merlin,. Tecla Pozzan, Lucio Sarto.

**Case Management Staff**: Andrea Brazzoli, Antonella Campi, Roberta Carmagnani, Sabrina Giambelli, Annalisa Gianella, Lino Lunardi, Davide Madaghiele, Paola Maestrelli, Lidia Paiola, Elisa Posteri, Loretta Viola, Valentina Zamberlan, Marta Zenari.

**Biological Sample processing and support to Brain Imaging precedures:** Sarah Tosato, Martina Zanoni, Giovanni Bonadonna, Mariacristina Bonomo.

**RESEARCH UNIT Eastern Veneto:**

**Coordinator:** Paolo Santonastaso.

**Administrative Leading Institution:** *University of Padua.*

**Coordinating Centre:** Carla Cremonese, Paolo Scocco, Angela Veronese.

**Participating MHCs: *TAU Arm****:* Ulss 8 ( Castelfranco), Ulss 9 (Treviso Nord; Oderzo), Ulss 10 (San Donà di Piave), Ulss 12 (Venezia; Mestre sud), Ulss 13 (Dolo), Ulss 14 (Piove di Sacco), Ulss 15 ( Cittadella), Ulss 16 (II° Servizio), Ulss 17 (Este; Montagnana).

***Experimental Arm:*** Ulss 8 (Montebelluna; Valdobbiadene), Ulss 9 (Treviso; Mogliano Veneto), Ulss 10 (PortogReserach Unitaro), Ulss 12 (Mestre Centro), Ulss 13 (Mirano), Ulss 14 (Chioggia I°; Cavarzere), Ulss 15 (Camposanpiero), Ulss 16 (I° Srvizio; III° Servizio), Ulss 17 (Monselice; Conselve).

**MHCs Reference Persons:** Patrizia Anderle, Andrea Angelozzi, Isabelle Amalric Gabriella Baron,, Enrico Bruttomesso Fabio Candeago, Franco Castelli, Maria Chieco, Carla Cremonese, Enrico Di Costanzo, Mario Derossi, Michele Doriguzzi, Osvaldo Galvano, Marcello Lattanzi, Roberto Lezzi, Marisa Marcato, Alessandro Marcolin, Franco Marini, Stefano Marino, Manlio Matranga, Elisabetta Sabbadin, , Rossana Riolo, Maria Zucchetto, Flavio Zadro.

**CBT Staff:** Daniela Argenti, Giovanni Austoni, Maria Bianco, Stefania Bordino, Linda Cibiniel, Maria Chieco, Marco Dall’Asta, Filippo Dario, Francesca Dassiè, Alessandro Di Risio, Aldo Gatto, Simona Granà, Emanuele Favero, Anna Franceschini, Silvia Friederici, Vanna Marangon, Marisa Marcato, Stefano Marino, Giorgio Martinelli, Michela Pascolo, Maya Piaia, Luana Ramon, Elisabetta Sabbadin, Paolo Scocco, Mara Semenzin, Angela Veronese, Stefania Zambolin, Maria Zucchetto. Anna Dominoni.

**Family Intervention Staff:** Antonella Buffon, Carla Cremonese, Elena Di Bortolo, Silvia Friederici, Stefania Fortin,Marisa Marcato, Francesco Matarrese, Simona Mogni, Novella Nicodemo, Alessio Russo, Alessandra Silvestro, Elena Turella, Paola Viel.

**Case Management Staff:** Lorenzo Andreose, Mario Boenco, Daniela Bottega, Loretta Bressan, Arianno Cabbia, Elisabetta Canesso, Romina Cian, Caludia Dal Piccol, Maria Dalla Pasqua, Cinzia De Gasperi, Anna Di Prisco, Lorena Mantellato, Monica Luison, Sandra Morgante, Mirna Santi, Moreno Sacillotto, Mauro Scabbio, Patrizia Sponga, MLuisa Sguotto, Flavia Stach, MGrazia Vettorato.

**Biological Sample processing and support to Brain Imaging precedures**: Oscar Cabianca, Amalia Valente, Livio Caberlotto, Alberto Passoni, Patrizia Flumian, Luigino Daniel, Massimo Gion, Saverio Stanziale, Flora Alborino, Vladimiro Bortolozzo, Lucio Bacelle, Leonarda Bicciato, Daniela Basso, Filippo Navaglia, Fabio Manoni, Mauro Ercolin.

**RESEARCH UNIT Emilia:**

**Coordinators:** Giovanni Neri, Franco Giubilini.

**Administrative Leading Institution:** *Azienda ULSS, Parma*

**Coordinating Centre:** Massimiliano Imbesi, Emanuela Leuci, Fausto Mazzi, Enrico Semrov.

**Participating MHCs:** ***TAU Arm***: Piacenza (Castel S.Giovanni), Parma (Parma Est; Sud Est; Valli Taro e Ceno), Reggio Emilia (CastelNovo nei Monti; Montecchio), Modena (Mirandola; Polo Ovest; Sassuolo; Pavullo).

***Experimental Arm***: Piacenza (Piacenza; Fiorenzuola), Parma (Nord; Ovest; Fidenza), Reggio Emilia (Correggio; Guastalla; Reggio Emilia III; Reggio Emilia; Scandiano), Modena ( Carpi; Polo Est; Vignola).

**MHCs Reference Persons**: Silvio Anelli, Mario Amore, Laura Bigi, Welsch Britta, Giovanna Barazzoni Anna, Rubes Bonatti, Maria Borziani, Stefano Crosato, Isabella Fabris, Raffaele Galluccio, Margherita Galeotti, Mauro Gozzi, Vanna Greco, Emanuele Guagnini, Stefania Pagani, Silvio Maccherozzi, Raffaello Malvasi, Francesco Marchi, Ermanno Melato, Elena Mazzucchi, Franco Marzullo, Pietro Pellegrini, Nicoletta Petrolini, Donatella Silvia Rizzi, Paolo Volta.

**CBT Staff:** Silvio Anelli, Franca Bonara, Elisabetta Brusamonti, Roberto Croci, Ivana Flamia, Francesca Fontana, Romina Losi, Fausto Mazzi, Roberto Marchioro, Stefania Pagani, Luigi Raffaini, Luca Ruju, Antonio Saginario, Giulia Stabili, Grazia Tondelli.

**Family Intervention Staff:** Lucia Bernardelli, Federica Bonacini, Annaluisa Florindo, Marina Merli, Patrizia Nappo, Lorena Sola, Ornella Tondelli, Matteo Tonna, MTeresa Torre, Morena Tosatti, Gloria Venturelli, Daria Zampolli.

**Case Management Staff**: Antonia Bernardi, Cinzia Cavalli, Lorena Cigala, Cinzia Ciraudo, Antonia Di Bari, Lorena Ferri, Fabiana Gombi, Sonia Leurini, Elena Mandatelli, Stefano Maccaferri, Mara Oroboncoide, Barbara Pisa, Cristina Ricci.

**Biological Sample processing and support to Brain Imaging precedures:** Enrica Poggi, Mara Oroboncoide, Corrado Zurlini, Monica Malpeli, Rossana Colla, Elvira Teodori, Luigi Vecchia, Rocco D'Andrea, Tommaso Trenti , Paola Paolini

**RESEARCH UNIT Romagna:**

**Coordinators:** Francesca Pileggi, Daniela Ghigi.

**Administrative Leading Institution:** *Azienda ULSS, Rimini*

**Coordinating Centre:** Mariateresa Gagliostro , Michela Pratelli, Paola Rucci

**Participating MHCs:** ***TAU Arm:*** Bologna (Zanolini; Scalo; Casalecchio; Vergato; San Giovanni), Ferrara (CSA Ferrara; SIPI Ferrara Sud; Codigoro; Portomaggiore), Ravenna (Ravenna; Fenza), Forlì (Forlì), Cesena (Cesena), Rimini (Riccione).

***Experimental Arm:*** Bologna (Mazzacorati; Tiarini, Nani; S. Lazzaro; Budrio; San Giorgio), Imola (UOT_Imola), Ferarra (Copparo; Ferrara Nord; Cento), Ravenna (Lugo), Cesena (Rubicone), Rimini (Rimini).

**MHCs Reference Persons**: Antonio Antonelli, Luana Battistini, Francesca Bellini, Eva Bonini, Caterina Bruschi Rossella Capelli, Cinzia Di Domizio, Chiara Drei, Giuseppe Fucci, Alessandra Gualandi, Maria Rosaria Grazia, Anna M. Losi, Franca Mazzanti Paola Mazzoni, Daniela Marangoni, Giuseppe Monna, Marco Morselli, Alessandro Oggioni, Silvio Oprandi, Walter Paganelli, Morena Passerini, Maria Piscitelli, Gregorio Reggiani, Gabriella Rossi, Federica Salvatori, Simona Trasforini, , Carlo Uslenghi, Simona Veggetti,

**CBT Staff:** Giovanna Bartolucci, Rosita Baruffa, Francesca Bellini, Raffaella Bertelli, Lidia Borghi, Patrizia Ciavarella, Cinzia DiDomizio, Giuseppe Monna, Alessandro Oggioni, Elisabetta Paltrinieri, Maria Piscitelli, Francesco Rizzardi, Piera Serra, Damiano Suzzi, Uslenghi Carlo.

**Family Intervention Staff:** Paolo Arienti, Fabio Aureli, Rosita Avanzi, Vincenzo Callegari, Alessandra Corsino, Paolo Host, Rossella Michetti, Michela Pratelli,Francesco Rizzo, Paola Simoncelli, Elena Soldati, Eraldo Succi.

**Case Management Staff:** Massimo Bertozzi, Elisa Canetti,Luca Cavicchioli, Elisa Ceccarelli, Stefano Cenni, Glenda Marzola, Vanessa Gallina, Carla Leoni, Andrea Olivieri, Elena Piccolo, Sabrina Ravagli, Rosaria Russo, Daniele Tedeschini.

**Biological Sample processing** **and support to Brain Imaging precedures**: Marina Verenini, Walter Abram, Veronica Granata, Alessandro Curcio, Giovanni Guerra, Samuela Granini, Lara Natali, Enrica Montanari, Fulvia Pasi, Umbertina Ventura, Stefania Valenti, Masi Francesca, Rossano Farneti, Paolo Ravagli, Romina Floris, Otello Maroncelli, Gianbattista Volpones, Donatella Casali.

**RESEARCH UNIT Firenze:**

**Coordinator:** Maurizio Miceli**.**

**Administrative Leading Institution:** *Azienda Sanitaria di Firenze*

**Coordinating Centre:** Maurizio Miceli**.**

**Participating MHCs: *TAU Arm:*** MOM SMA 5; MOM SMA 8; MOM SMA 11; MOM SMA 12.

***Experimental Arm:*** MOM SMA 3; MOM SMA 7; MOM SMA 9; MOM SMA 10.

**MHCs Reference Persons*:*** Andrea Bencini, Massimo Cellini, Luca De Biase, Leonardo Barbara, Liedl Charles, Maurizio Miceli, Cristina Pratesi, Andrea Tanini, Roberto Leonetti.

**CBT Staff:** Massimo Cellini, Maurizio Miceli, Riccardo Loparrino, Cristina Pratesi, Cinzia Ulivelli,

**Family Intervention Staff*:*** Cristina Cussoto, Nico Dei, Enrico Fumanti, Manuela Pantani, Gregorio Zeloni.

**Case Management Staff:** Rossella Bellini, Roberta Cellesi, Nadia Dorigo, Patrizia Gullì, Luisa Ialeggio, Maria Pisanu.

**Biological Sample processing and support to Brain Imaging precedures:** Graziella Rinaldi, Angela Konze

**RESEARCH UNIT Milano Niguarda:**

**Coordinator:** Angelo Cocchi**.**

**Administrative Leading Institution:** Azienda Ospedaliera Ospedale Niguarda Ca’ Granda*, Milano*

**Coordinating Centre:** Anna Meneghelli

**Participating MHCs: *TAU Arm:*** corso Plebisciti; via Mario Bianco.

***Experimental Arm:*** via Cherasco e via Livigno; via Litta Modignani.

**MHC Reference Persons:** Maria Frova , Emiliano Monzani, Alberto Zanobio, Marina Malagoli, Roberto Pagani.

**CBT Staff:** Simona Barbera, Carla Morganti, Emiliano Monzani, Elisabetta Sarzi Amadè.

**Family Intervention Staff**: Virginia Brambilla, Anita Montanari.

**Case Management Staff:** Giori Caterina, Carmelo Lopez.

**Biological Sample processing and support to Brain Imaging precedures:** Alessandro Marocchi, Andrea Moletta**,** Maurizio Sberna

**RESEARCH UNIT Milano S. Paolo:**

**Coordinator:** Silvio Scarone.

**Administrative Leading Institution:** *Azienda ULSS San Paolo, Milano*

**Coordinating Centre:** Maria Laura Manzone

**Participating MHCs: *TAU Arm*:** CPS Zona 14 (Barabino).

***Experimental Arm:*** Rozzano; Zona 15 (Conca del Naviglio); Zona 16 (San Vigilio).

**MHC Reference Persons:** Barbera Barbara, Luisa Mari, Maria L. Manzone, Edoardo Razzini.

**CBT Staff:** Yvonne Bianchi, MRosa Pellizzer, Antonella Verdecchia.

**Family Intervention Staff:** MGabriella Sferrazza, MLaura Manzone, Carmine Pismataro.

**Case Management Staff:** Benedetta Cerrai, Alessandra Gambino, Rosa Panarello.

**Biological Sample processing and support to Brain Imaging precedures:** Gian Vico Melzi D'Eril, Alessandra Barassi, Rosana Pacciolla, Gloria Faraci

**RESEARCH UNIT Bolzano:**

**Coordinator:** Stefano Torresani (Bolzano).

**Administrative Leading Institution:** *Azienda Sanitaria,, Bolzano*

**Participating MHCs:** ***TAU Arm:*** none

***Experimental Arm:*** Bolzano Rossini; Bolzano del Ronco.

**MHC Reference Persons:** Fabio Carpi, Soelva Margit.

**CBT Staff:** Monica Anderlan, Michele De Francesco, Efi Duregger, Stefano Torresani, Carla Vettori.

**Family Intervention Staff:** Carpi Fabio, Doimo Sabrina, Kompatscher Erika, Soelva Margit, Stefano Torresani

**Case Management Staff:** Forer Michael, Kerschbaumer Helene.

**Biological Sample processing and support to Brain Imaging precedures:** Anna Gamper, Maira Nicoletti

**Psychotherapists supporting treatments in the experimental arm:**

Chiara Acerbi, Daniele Aquilino, Silvia Azzali, Luca Bensi, Sarah Bissoli, Davide Cappellari, Elisa Casana, Nadia Campagnola, Elisa Dal Corso, Elisabetta Di Micco, Erika Gobbi, Laura Ferri, Erika Gobbi, Laura Mairaghi, Sara Malak, Luca Mesiano, Federica Paterlini, Michela Perini, Elena Maria Puliti, Rosaria Rispoli, Elisabetta Rizzo, Chiara Sergenti, Manuela Soave, Elisabetta Di Micco, Rosaria Rispoli.

**Experts supervising treatments in the exprerimental arm:**

Andrea Alpi, Laura Bislenghi, Tiziana Bolis, Francesca Colnaghi, Simona Fascendini, Silvia Grignani, Anna Meneghelli, Giovanni Patelli.

**SPECIFIC TOPICS RESEARCH UNITS:**

**RESEARCH UNIT Life Events Firenze - Coordinator:** Carlo Faravelli

**Coordinating Centre:** Silvia Casale

**Administrative Leading Institution:** *University of Florence*

**RESEARCH UNIT Communications Skills - Coordinator:** Christa Zimmermann

**Coordinating Centre:** Giuseppe Deledda, Claudia Goss, Mariangela Mazzi, Michela Rimondini.

**Administrative Leading Institution:** *University of Verona*

**RESEARCH UNIT Genetics**-IRCCS, FBF Brescia – **Coordinator:** Massimo Gennarelli

**Coordinating Centre:** Catia Scassellati, Cristian Bonvicini, Sara Longo

**Administrative Leading Institution:** IRCCS Centro S.Giovanni di Dio Fatebenefratelli, Brescia

**RESEARCH UNIT Neuropsicopharmacology-**IRCCS, FBF Brescia – **Coordinator:** Luisella Bocchio Chiavetto

**Coordinating Centre:** Roberta Zanardini

**Administrative Leading Institution:** IRCCS Centro S.Giovanni di Dio Fatebenefratelli, Brescia

**RESEARCH UNIT Molecular Biology**, AFaR, FBF, Roma – **Coordinator:** Mariacarla Ventriglia

**Coordinating Centre:** Rosanna Squitti

**Administrative Leading Institution:** Department of Neuroscience, AFaR-Fatebenefratelli Hospital, Rome, Italy

**RESEARCH UNIT LENITEM** - IRCCS, FBF Brescia – **Coordinator:** Giovanni Frisoni

**Coordinating Centre:** Michela Pievani

**Administrative Leading Institution:** IRCCS Centro S.Giovanni di Dio Fatebenefratelli, Brescia

**RESEARCH UNIT RUBIN**, Udine-Verona – **Coordinator:** Matteo Balestrieri

**Coordinating Centre:** Paolo Brambilla, Cinzia Perlini, Veronica Marinelli, Marcella Bellani, Gianluca Rambaldelli, Alessandra Bertoldo, Paolo Carpeggiani, Alberto Beltramello, Franco Alessandrini, Francesca Pizzini, Giada Zoccatelli, Maurizio Sberna, Angela Konze

**Administrative Leading Institution:** DISM, Università di Udine, Udine (Signora Marina Dorligh)

**RESEARCH UNIT STRESS,** University of Pavia – **Coordinator:** Pierluigi Politi

**Coordinating Centre:** Enzo Emanuele, Natascia Brondino.

**RESEARCH UNIT Neuroimmunologiy**-IRCCS S. Raffaele, Milano – **Coordinator:** Gianvito Martino

**Coordinating Centre:** Alessandra Bergami e Roberto Zarbo

**RESEARCH UNIT Animal Models**, Univ. Milano – **Coordinator:** Marco Andrea Riva

**Coordinating Centre:** Fabio Fumagalli, Raffaella Molteni, Francesca Calabrese, Gianluigi Guidotti, AlessiaLuoni, Flavia Macchi.

**Independent evaluators and researchers supporting the onsite data collection**:

Stefania Artioli, Marco Baldetti, Milena Bizzocchi, Donatella Bolzon, Elisa Bonello, Giorgia Cacciari, Claudia Carraresi, MTeresa Cascio, Gabriele Caselli, Karin Furlato, Sara Garlassi, Alessandro Gavarini, Silvia Lunardi, Fabio Macchetti, Valentina Marteddu, Giorgia Plebiscita, Sara Poli, Stefano Totaro.

**FAMILIES AND USERS ASSOCIATION (AITSAM):**

Tali Corona Mattioli

**INTERNATIONAL ADVISORY BOARD:**

PIANO: Paul Bebbington, Max Birchwood, Paola Dazzan, Elisabeth Kuipers, Graham Thornicroft;

GUITAR: Carmine Pariante; CONTRABASS: Steve Lawrie, Carmine Pariante, Jair C. Soares
